# Control of polarized assembly of actin filaments in cell motility

**DOI:** 10.1007/s00018-015-1914-2

**Published:** 2015-05-07

**Authors:** Marie-France Carlier, Julien Pernier, Pierre Montaville, Shashank Shekhar, Sonja Kühn, Cytoskeleton Dynamics and Motility group

**Affiliations:** Institut de Biologie Intégrative de la Cellule, CNRS, 91198 Gif-sur Yvette, France

**Keywords:** Actin filament barbed end, Treadmilling, Processivity, Formins, WH2 domains, WASP family proteins, Arp2/3 complex, Filament branching, Capping proteins, Profilin, Cell protrusion

## Abstract

Actin cytoskeleton remodeling, which drives changes in cell shape and motility, is orchestrated by a coordinated control of polarized assembly of actin filaments. Signal responsive, membrane-bound protein machineries initiate and regulate polarized growth of actin filaments by mediating transient links with their barbed ends, which elongate from polymerizable actin monomers. The barbed end of an actin filament thus stands out as a hotspot of regulation of filament assembly. It is the target of both soluble and membrane-bound agonists as well as antagonists of filament assembly. Here, we review the molecular mechanisms by which various regulators of actin dynamics bind, synergize or compete at filament barbed ends. Two proteins can compete for the barbed end via a mutually exclusive binding scheme. Alternatively, two regulators acting individually at barbed ends may be bound together transiently to terminal actin subunits at barbed ends, leading to the displacement of one by the other. The kinetics of these reactions is a key in understanding how filament length and membrane-filament linkage are controlled. It is also essential for understanding how force is produced to shape membranes by mechano-sensitive, processive barbed end tracking machineries like formins and by WASP-Arp2/3 branched filament arrays. A combination of biochemical and biophysical approaches, including bulk solution assembly measurements using pyrenyl-actin fluorescence, single filament dynamics, single molecule fluorescence imaging and reconstituted self-organized filament assemblies, have provided mechanistic insight into the role of actin polymerization in motile processes.

## Introduction

The actin cytoskeleton is a dynamic architecture of the living cell, made of several structurally and functionally distinct arrays of actin filaments that define modular motile activities. Nucleation and polarized assembly of actin filaments are driven locally in a stimulus-responsive fashion in each module by specific protein machineries. These reactions develop forces of compression or traction against the membranes to elicit protrusive, adhesive and contractile activities ([1] for review). Dendritic arrays of branched filaments are assembled at the leading edge of lamellipodia, at the neck of endocytic vesicles, in podosomes. Linear actin bundles are arranged in parallel fashion in filopodia and microspikes within lamellipodia, and in antiparallel fashion in contractile stress fibers. Cells thus use actin to move, feed, divide, and organize intracellular traffic. These actin-based machineries are also harnessed by intracellular pathogens to propel themselves and facilitate their propagation ([2] for review).

A large number of experiments have established that the dynamic nature of the actin cytoskeleton is essential for motility. Movement is abolished if cells are treated with drugs that either depolymerize or stabilize actin filaments. Actin filament polarized assembly in a motile cell has been demonstrated by a number of live-cell imaging methods such as fluorescence recovery after photobleaching (FRAP), fluorescence loss in photobleaching (FLIP), fluorescence localization after photobleaching (FLAP), and fluorescence speckle microscopy (FSM) [3, 4]. To gain a fundamental understanding of cell motility, it is essential to understand how actin assembly is spatially and kinetically maintained and controlled in cells.

**Fig. 1:**
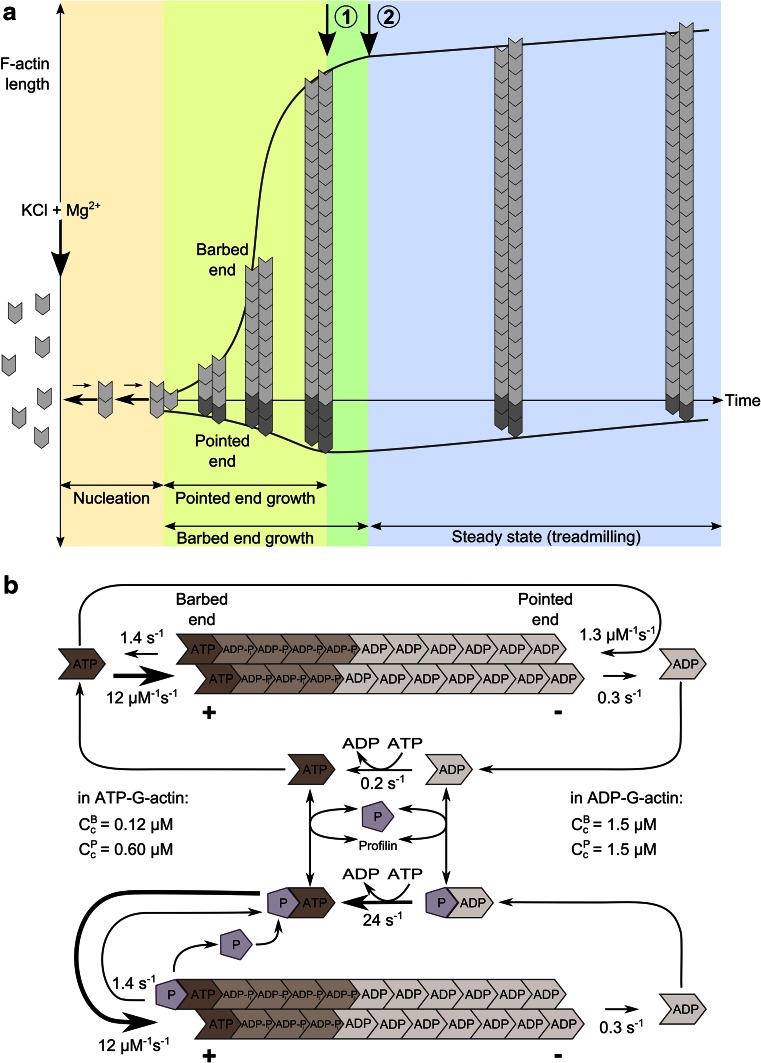
Diagram of self-assembly of an “average” actin filament in vitro. **a** Spontaneous assembly of ATP-actin in vitro is initiated by a sudden increase in ionic strength at time zero, in a solution of actin monomers (G-actin). Nucleation is followed by endwise association of G-actin molecules to nuclei, faster at the barbed end than at the pointed end. As F-actin is assembled, the concentration of G-actin monomers decreases in solution. Pointed end growth rate reaches zero (*arrow 1*) when the concentration of G-actin reaches the critical concentration for pointed end assembly (0.6 µM). Barbed end growth goes on and G-actin concentration declines, while pointed ends start to disassemble. When the steady-state concentration of G-actin (0.1 µM) is reached (*arrow 2*), equal net rates of barbed end assembly and pointed end disassembly (treadmilling) maintain a constant amount of F-actin in solution, schematized here by a constant length of the “average” filament. **b** Nucleotide hydrolysis associated with the treadmilling cycle of the actin filament at steady-state in ATP, with and without profilin. The barbed end terminal subunits are enriched in ATP/ADP-Pi, while ADP is bound to pointed end terminal subunits. Profilin–ATP-actin participates in barbed end assembly, but not in pointed end assembly, hence it enhances processivity of treadmilling. In the cellular medium, treadmilling is regulated to generate variable rates of barbed end assembly. Regulation is performed either by increasing the rate limiting step of the treadmilling cycle which is pointed end depolymerization (using ADF/cofilin), or by regulating the dynamics at barbed ends (capping, tracking, destabilizing)

In a living cell, actin is in a dynamic equilibrium (or rather steady-state) between two states—the globular monomeric state (G-actin) and the polymeric filament state (F-actin). At physiological ionic strength, actin is essentially polymerized in filaments (F-actin). Movement is intimately linked to actin exchanges between these two states. These exchanges are dissipative due to ATP hydrolysis that is associated with actin assembly ([5] for review). This is why in a living cell actin is assembled at a steady-state, in contrast to equilibrium polymers assembled by reversible association–dissociation reactions. Because the assembly–disassembly kinetics are faster at the barbed ends than at the pointed ends as compared to the rate of ATP hydrolysis, terminal actin subunits at barbed ends are mostly ATP or ADP-Pi bound, while ADP-actin is mainly exposed to the pointed end (Fig. 1a, b). The resulting energetic bias in monomer–polymer exchanges at the two ends generates a net flux of subunits from one end to the other, called treadmilling, which is intrinsically extremely slow for pure actin (See Fig. 1 for detailed information). It is therefore only via the regulation of treadmilling by actin-binding proteins that fast polarized actin assembly can occur in cells.

The relative sizes of the assembled and unassembled actin pools are regulated in several ways, which are interconnected. The mechanically simplest regulators are G-actin sequestering proteins. Proteins such as β-thymosins bind G-actin in a complex that does not assemble in filaments. This pool of sequestered actin is in rapid equilibrium with only G-actin, not with F-actin, and cannot be invoked to support actin assembly directly. It can only amplify the changes in amount of F-actin that result from changes in stationary concentration of free monomers, elicited by regulators of assembly dynamics at filament barbed ends. In contrast with G-actin sequestering proteins, profilin binds G-actin in a complex that participates in barbed end assembly specifically (Fig. 1b), thus enhancing the processivity of treadmilling.

Live-cell imaging of actin filament dynamics in lamellipodia of migrating cells indicates that filaments treadmill at constant rate as the leading edge moves forward [3, 4]. Similarly, pathogens like *Listeria monocytogenes*, which exploit actin-based motility, move in the cytoplasm at constant rates for long periods of time [6, 7]. These features indicate that movement is mediated by the steady-state turnover of actin filaments. They are incompatible with other evoked mechanisms such as a sudden increase in availability of large amounts of G-actin [1], which would generate sudden transient movements that would exponentially slow down to arrest upon reaching steady-state, as observed in a test tube when G-actin is induced to assemble at time zero.

In the cellular context, fundamental questions regarding the mechanism of production of force and movement by actin polymerization remain unanswered. For polarized growth of filaments to be maintained at defined sites in the cell, e.g., at the protein–membrane interface, some transient attachment of the growing barbed ends appears to be required. What structural and functional mechanisms are able to locally restrain and control filament growth? How are these chemical reactions at the interface of filaments and membranes transduced into mechanical properties, at the molecular and supramolecular scale? What laws of physical chemistry of protein self-assembly account for coordinated filament turnover in various arrays and for actin homeostasis? If dendritic arrays are assembled using the same basic molecules in different motile functions, how does the cell avoid indirect effects resulting from one motile activity (e.g. endocytosis) on the efficiency of other modules? Answering these questions requires a detailed understanding of the underlying molecular mechanisms at high spatial and temporal resolution. Integrated interdisciplinary approaches have shown promise of providing answers at different scales to these burning issues.

## The barbed end of the actin filament: a hotspot of actin assembly regulation

**Fig. 2:**
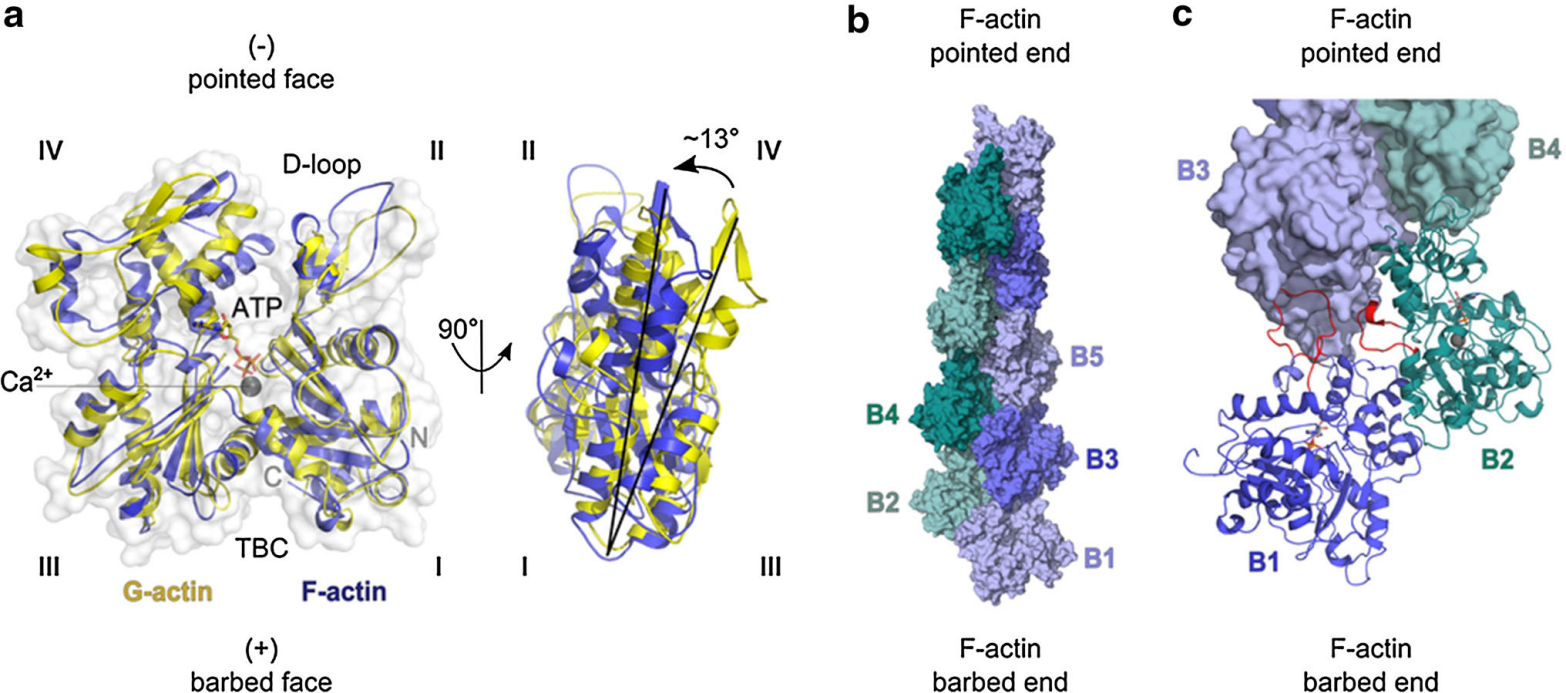
Structures of G-actin and F-actin filament barbed ends. **a** The transition of G- to F-actin. The structures of actin in the globular G-actin (*yellow*) or in the filamentous F-actin (*blue*) state are superimposed. They originate from G-actin in complex with DNAse I with bound Ca^2+^-ion and ATP (1ATN; [122]) and F-actin (2ZWH; [123]). The DNAse I-binding loop (D-loop) and subdomains *I*–*IV* are labeled. The target binding cleft (TBC) at the barbed face of actin is located between subdomains *I* and *III*. Actin protomers are flattened in F-actin by a 13° twist of the outer subdomains (*I* and *II*) to the inner ones (*III* and *IV*). **b** Surface representation of the double-helical structure of a 167° twisted F-actin nonamer (4A7N; [124]). The fast growing barbed end and slow growing pointed end are indicated. **c** Important longitudinal contacts between terminal F-actin subunits at the filament barbed end. The two terminal actin protomers B1 and B2 are depicted as cartoon. Loops involved in intermolecular binding are highlighted (*red*). The D-loop of actin B1 (aa 31–51) including its adjacent C-terminal region (aa 61–65) binds into the TBC of protomer B3. It also contributes to the transverse interaction between loop aa 265–271 of B2 with B3. The longitudinal contact of loop aa 243–245 of B1 with B3 is not visible in this representation [9, 123]

An actin filament is a chiral helical polymer in which all actin subunits share the same polarity, defining a “barbed end” and a “pointed end” (Figs. 1b, 2). A large number of proteins control actin dynamics by binding the barbed face of actin. The barbed face of actin is exposed on G-actin and also on the two terminal subunits (the ultimate and penultimate protomers, at positions B1 and B2 in Fig. 2c) of the filament barbed end. This feature introduces several levels of potential complexity in the regulation.

First, the two terminal subunits are subject to weaker bonding constraints compared to the actin subunits further in the core of the filament, which are connected via two lateral and two longitudinal contacts. The acknowledged structural plasticity of the filament core [8] is potentially enhanced at the barbed ends, which might be exploited by regulators to generate a large variety of functional states. The actual structure of these two subunits is not known, but they might adopt a conformation closer to that of G-actin (Fig. 2a) than to the F-actin subunits in the core of the filament in which the subdomains twist into a “flatter” structure (Fig. 2b). “Primary” regulators that interact with the terminal actin subunits at the barbed end may either facilitate or prevent further binding of “secondary” regulators, thereby building up structurally distinct filament arrays. The nucleotide state of the terminal subunits i.e., ATP, ADP-Pi or ADP may modulate the affinity of various regulators for the barbed end of the filament, potentially increasing the complexity of the regulation.

**Fig. 3:**
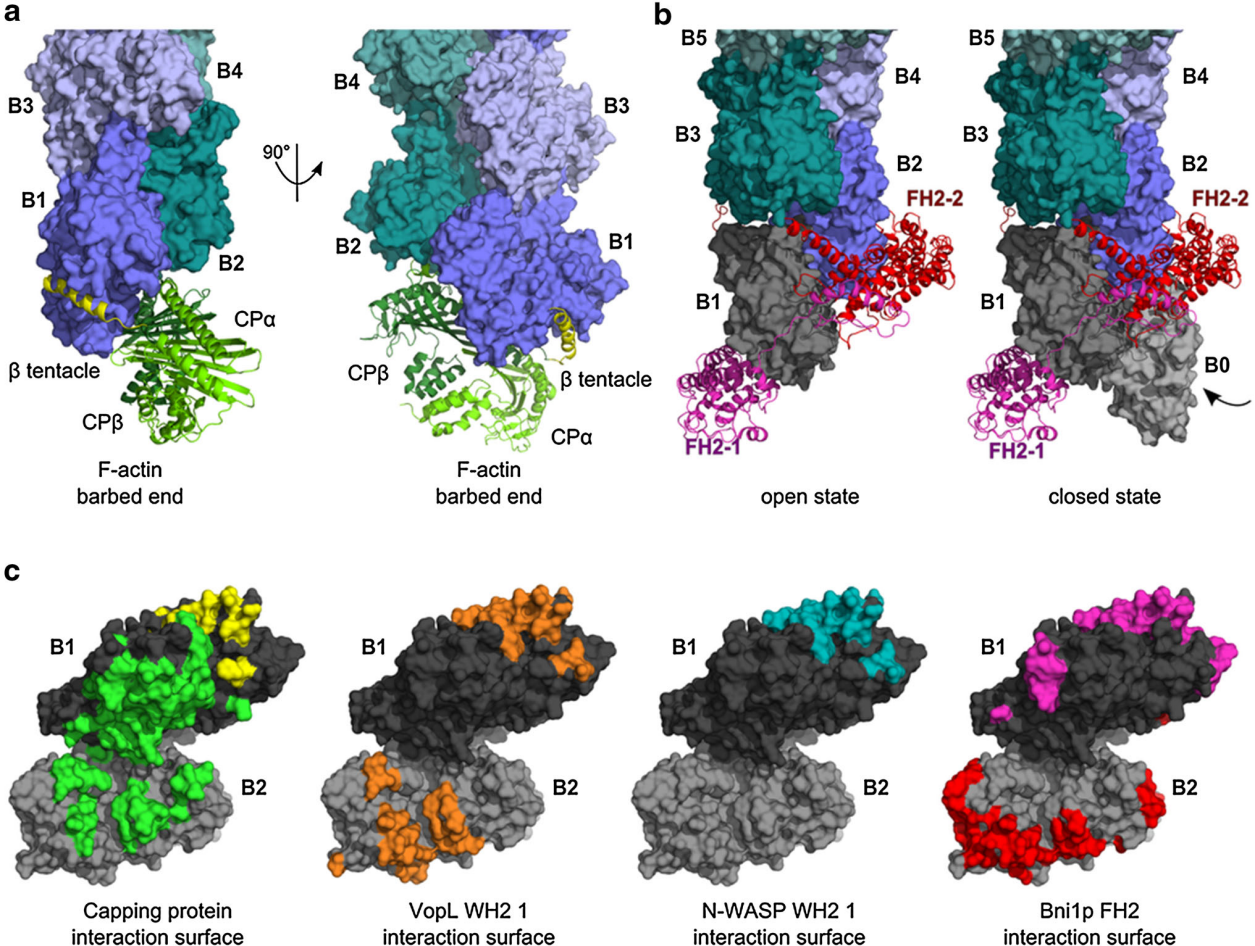
Structure of actin regulators bound to the barbed end. **a** Structure of CP bound to the barbed end. The α/β heterodimeric capping protein (CP) is illustrated in *ribbon diagrams* (CPα: *light green*; CPβ: *green*; [43]; coordinates kindly provided by Y. Maeda). CPαβ forms strong electrostatic interactions at the interface of B1 and B2, while CPβ binds with its amphipathic β-tentacle (βT, *yellow*) to the hydrophobic TBC of B1. **b** Structure of a dimeric formin homology 2 (FH2) domain at the barbed end. The crystal structure of the FH2 domain of yeast formin Bni1p was crystallized encircling a flattened, 180° twisted pseudo filament (1Y64; [54]). The Bni1-FH2/G-actin structure was superimposed on actin B2 of the 167° twisted F-actin barbed end (4A7N, shown in *green*–*blue*). The 180° twisted protomers B1 and B0 are depicted in *grey*. The amphipathic α-helix of the knob region of each FH2 hemidimer (chains FH2-1, FH2-2; *magenta*, *red*) binds to the TBC of B1 and B2, respectively. **c** Interaction surface of actin regulators at the barbed end. Highlighted residues of B1 (*dark grey*) and B2 (*grey*) are involved in binding to the various regulators. Many barbed end binding proteins associate with an α-helix (e.g. β-tentacle of CP, *yellow* surface) to the TBC of actin and additionally with other surface areas specific for each interaction. Surface coloring: residues of the barbed end involved in binding to CP (CPαβ *green*, β-tentacle *yellow*), VopL-WH2 1 (3M1F, *orange*, aa 130–151; [125]), N-WASP-WH2 1 (3M3N, *blue*, aa 397–418; [125]), or Bni1p-FH2 domain (1Y64, *magenta*, *red*; [54]). Since VopL dimerizes, the interacting residues of the first, N-terminal WH2 domain of each VopL chain were highlighted on B1 and B2, respectively. The diagram suggests that two barbed end binding regulators can bind together to B1 and B2 pending some loss of binding strength, and use this transient ternary complex to displace each other. Examples include uncapping of CP by VopF [21] and by formin (Shekhar et al., submitted)

Second, barbed end binding proteins may associate either with only one or both actins at the barbed ends (Fig. 3). Occupancy of the barbed face by a regulator leaves the barbed face of the penultimate subunit available for interaction with other regulators. On terminal actin subunits, binding regions are shared by several regulators and additional binding regions are specific to individual regulators. Thus, complexes in which two regulators are bound together at filament barbed ends are potentially formed, pending lowered affinity (Fig. 3). This may lead to far more complex mechanistic schemes for barbed end regulation than the simple models merely relying on mutually exclusive binding.

A frequently used binding site, targetted by actin-binding proteins as well as by drugs like macrolides, is the hydrophobic pocket in the shear zone between subdomains 1 and 3 also called target binding cleft, TBC (Figs. 2a, 3). Amphipathic α-helices of ß-thymosins, WH2 domains, gelsolin, formins, twinfilin, capping protein (CP), Eps8, RPEL motif of MAL proteins, etc. with 3–5 turns, irrespective of their polarity, dock in this pocket ([9], for review). The strength of binding of this α-helix in itself does not strictly correlate with a defined function. To be specific, ß-thymosins and RPEL motifs sequester G-actin and inhibit actin polymerization. In contrast, some WH2 domains bind G-actin as functional homologs of profilin, thus facilitating barbed end assembly. Gelsolin, CP, Eps8, twinfilin, Cytochalasin D and other macrolides cap barbed ends, while formins and some WH2 domains either cap or track barbed ends. Table 1 summarizes the structure–function relationships and modes of action of these various regulators of barbed end assembly.

**Table 1:** Regulators of actin filament barbed end dynamics and their specific mode of action

Main activity	Regulator	Specific mode of function
Participation in barbed end assembly	Profilin	Binds G-actin with high affinity Profilin–actin supports exclusively barbed end growth Profilin–actin is substrate for formins Binds barbed ends with low affinity and enhances disassembly
	WASP proteins (WH2)	Catalyze filament branching with Arp2/3 complex Capture barbed ends via WH2 domain Facilitate association of bound G-actin to barbed ends via WH2 domain (profilin-like activity)
	Cordon-Bleu (WH2)	Facilitates association of bound G-actin to barbed ends Nucleates and severs actin filaments
	ADF/cofilin	Increases depolymerisation of pointed ends, causing an increase in pointed end critical concentration which in turn leads to enhanced barbed end assembly at steady-state
Barbed end tracking	Ena/VASP (WH2)	Processively elongate F-actin barbed ends, promote dissociation of barbed end assembly antagonists (uncapping)
	VopF/VopL (WH2)	Track F-actin barbed ends, promote dissociation of barbed end assembly antagonists (uncapping)
	Formins (FH1–FH2)	Nucleate actin filaments, catalyse rapid processive BE assembly from profilin–actin
Barbed end capping	Group I: Gelsolin, villin, brevin, severin, adseverin (very high affinity for barbed end, *K*_F_ = 10^−11^M)	Prevent spontaneous nucleation and barbed end growth. Maintain a large pool of unassembled actin Sever and cap filaments
	Group II: CP, Esp8, CapZ, twinfilin, IQGQP1, CapG (lower affinity binding to barbed end, *K*_F_ = 10^−9^M)	Prevent spontaneous nucleation and barbed end growth. Maintain a large pool of unassembled actin
	Spire (WH2)	Caps barbed ends weakly, preventing growth from profilin–actin Severs and caps filaments Recruits Formin 2 to actin filament barbed end

Most of these effectors have been characterized, using bulk solution assays and single filament assembly dynamics. Importantly, in cellular conditions, some barbed end regulators are soluble and free in the cytoplasm; others act in a membrane-bound state. The interplay between soluble and immobilized regulators of filament barbed end assembly drives the dynamic coupling between the actin cytoskeleton and membranes (or vesicles), and is pivotal in force production and shape definition. The functional competition between agonists and antagonists of barbed end growth raises structural and mechanistic issues.

## Cytoplasmic soluble agonists and antagonists of filament barbed end assembly

### Soluble agonists of polarized actin assembly: direct and indirect mechanisms

The most important agonist of barbed end assembly is profilin. Profilin is a ubiquitous protein, present at concentrations in the range of 10–100 µM in cells. As a result of its binding to the barbed face of actin, it displays a dual interaction with G-actin and terminal subunits at filament barbed ends (Fig. 1b). The complex of profilin with ATP-G-actin supports filament growth at barbed ends exclusively with the same kinetic parameters as free G-actin [10]. Profilin–actin acts as an effective substrate of fast processive assembly catalyzed by formins [11–13]. Notably, in binding to barbed end terminal subunits, profilin destabilizes actin–actin contacts and enhances the rate of actin dissociation from the barbed end [14, 15]. Profilin also competes with other barbed end binding proteins, in particular, it antagonizes capping proteins (Pernier et al., submitted).

WH2 domains are intrinsically disordered actin-binding modules that fold upon binding to actin, sharing the binding mode of β-thymosins. However, with a similar structural motif, they developed a large panoply of versatile functions different from β-thymosins. The WH2 domains in themselves are actin-binding blocks whose regulatory functions in actin assembly are governed by the electrostatic environment in which they bind  actin [16]. Some WH2 domains behave as functional homologs of profilin, facilitating association of the bound actin to filament barbed ends: the WH2 domain of WASP proteins and the Cordon-Bleu protein, are known examples. Some WH2 domains facilitate nucleation by binding the actin monomer and neutralizing its negative charge, thus enhancing formation of prenuclei dimers. While tandem repeats of WH2 domains present in Spire, Cordon-Bleu or the pathogen effectors VopF and VopL display this nucleating function, a single WH2 domain of Cordon-Bleu, flanked by a lysine-rich short extension, is sufficient to nucleate actin [17]. WH2 domains also may directly bind and track the terminal subunits at barbed ends, allowing association of G-actin and filament growth, in competition with other barbed end binding proteins. The WH2 domains of Spire cap barbed ends [18–20]; those of VopF or of Ena/VASP use their dimeric quaternary structure to track barbed ends and promote rapid dissociation of bound CP, a reaction called “uncapping” [21].

ADF/cofilin is another essential soluble regulator, which specifically binds ADP-bound G- and F-actin. Hence, it does not interact with ATP-bound growing filament barbed ends, yet it facilitates barbed end assembly in a paradoxical fashion, as follows: ADF/cofilin destabilizes filaments structurally and thermodynamically by weakening interactions between ADP–F-actin subunits. This results in a large increase in the intrinsic depolymerization rate constant of ADP-bound pointed ends, which promotes a large increase in the steady-state concentration of monomeric ATP-actin. This is confirmed by in vivo experimental evidence where higher concentration of monomers promotes faster polymerization at barbed ends ([22] for review).

### Soluble antagonists of filament barbed end assembly

Barbed end capping proteins block barbed end assembly. They are generally cytoplasmic and act in soluble form, but might be immobilized in specific loci by regulatory ligands like CARMIL [23, 24]. A large variety of capping proteins is found in living cells. They differ in their abundance, structure and strength of barbed end binding. Gelsolin and its related proteins (severin, adseverin, villin, brevin) bind extremely tightly to barbed ends, with binding constants of the order of 10^−11^ M [25, 26]. Other cappers such as capping protein, the most ubiquitous and abundant one (about 1–2 µM in cells [27]), its muscle homolog CapZ, Eps8 [28], twinfilin [29], IQGAP1 [30], or Ca^2+^-dependent CapG which mimics a half-gelsolin molecule [31], display affinities lower than gelsolin i.e., binding constants to barbed ends in the subnanomolar to nanomolar range. Some WH2 repeat proteins such as Spire cap barbed ends with nanomolar affinity. An increasing body of evidence shows that capping proteins assist in maintaining a large enough pool of unassembled polymerizable actin monomers that is used locally and transiently for barbed end growth and protrusive force e.g., in lamellipodia or filopodia [32]. Capping proteins are also useful to block unnecessary filament nucleation in the cytoplasm. The balance between soluble cappers and membrane-immobilized nucleating factors thus maintains polarized actin assembly. This point is further developed in the next section.

## Filament turnover and barbed end nucleation and growth are chemically coupled

The maintenance of a stationary concentration of monomeric polymerizable actin, which supports sustained local nucleation and polarized growth of filaments is made possible if a large enough fraction of the population of filament barbed ends are capped, thus setting the high concentration of monomeric actin imposed by pointed end dynamics [33]. The available monomeric actin can then be “funneled” towards the remaining non-capped filaments. Consistently, loss of CP slows down cell motility, while its overexpression enhances it [3, 34] and CP is required for cell migration [32].

**Fig. 4:**
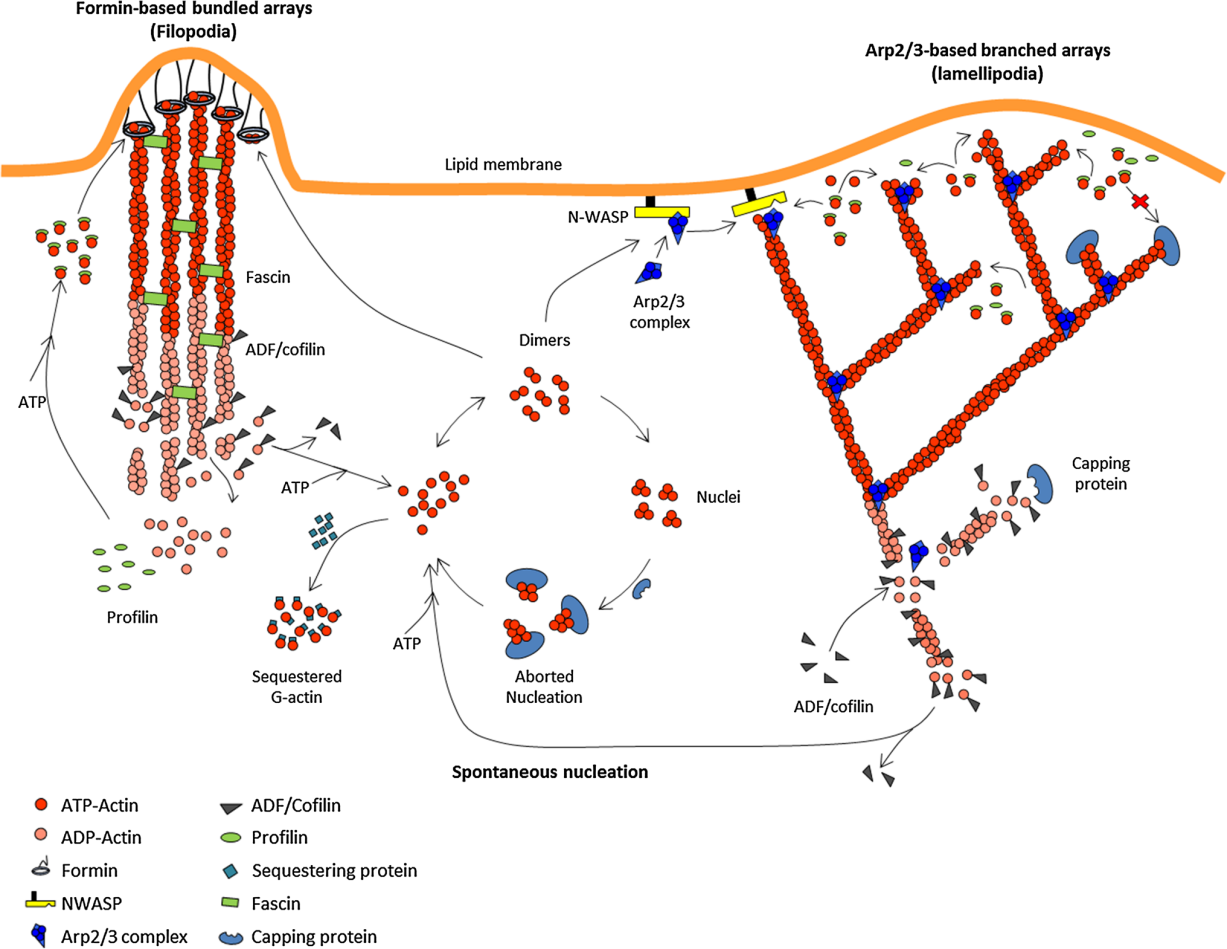
Sketch of the regulation of filament assembly in motile processes. Regulated treadmilling drives both site-directed barbed end nucleation and polarized assembly. For simplicity, only the protein machineries responsible for filament branching (WASP family proteins) and for processive individual filament assemblies (formins) are drawn. Filament tracking by Ena/VASP and other WH2 domain proteins are conceptually similar, and not shown for simplicity. In the generalized treadmilling cycle, polymerizable ATP-bound actin monomers are produced by depolymerization of ADP-actin from filament ADP-bound pointed ends, facilitated by ADF/cofilin. Note that an excess of ADF will block monomers in the ADF–ADP-bound non-motile state (no treadmilling), because nucleotide exchange is inhibited by ADF. Thus, the effect of ADF on motility presents a *bell shape* dependence on concentration. Spontaneous nucleation by ATP-actin is aborted in cytoplasm by capping protein, and locally facilitated by nucleators. Formin-induced nucleation requires actin dimers. The sketch implicitly assumes that an actin dimer/trimer prenucleus can as well undergo branching with WASP and Arp2/3 complex. Capping protein arrests filament growth in dendritic filament arrays. A balanced number of filament barbed ends is maintained via the equal frequency of “birth” by branching and “death” by capping. Capping protein is also required for regulating the length of formin-induced filament in filopodia

Capping proteins thus act in synergy with actin depolymerizing factor, which causes an increase in the critical concentration at the pointed ends by enhancing the rate of pointed end disassembly. The resulting higher stationary concentration of actin monomers facilitates spontaneous nucleation. Spontaneously formed nuclei abort in the cytoplasm by their association with capping proteins, but are stabilized locally by association with membrane-bound nucleators (Fig. 4). Thus, in an apparent paradoxical fashion, agents that destabilize filaments and block barbed end growth in the cytoplasm actually facilitate locally stimulated creation of new filaments and faster barbed end growth of filaments at the individual level. These filaments grow for a short period of time, because CP eventually blocks their growth. These effects have been verified in vitro in reconstituted motility of N-WASP-coated and formin-coated beads, which both propel faster in the presence of a minimal amount of CP in the motility medium [13, 35]. In vivo as well, the indirect effect of capping proteins on the growth of either free or formin-bound (or VASP-bound) barbed ends identically accounts for faster lamellipodia and filopodia extension in the presence of capping proteins. Consistently, slow filopodial dynamics are observed in CP-depleted cells [32]. Note that CP-depleted cells display an increased amount of F-actin because the imposed lower value of the critical concentration for filament assembly also imposes a lower amount of sequestered actin; nonetheless, these cells move slowly, indicating that the speed of actin-based movement does not increase with the amount of assembled actin, but with the stationary amount of polymerizable monomers.

Along the same line of logic, overexpression of soluble, constitutively active protein machineries that promote filament barbed end assembly, like the catalytic domain VCA of WASP proteins, or constitutively active formin fragments, should functionally antagonize capping proteins by imposing filament barbed end dynamics in the cytoplasm and causing a massive assembly of actin filaments similar to the phenotype of capping protein deletion. The resulting imposed lower concentration of polymerizable monomeric actin impairs migration and all motile processes. A different phenotype is generated by overexpressing the CA fragment of WASP proteins or any CA-related protein. These do not activate Arp2/3 complex but sequester it, hence they impose a lower limit of filament branching by insufficient amount of substrate. In conclusion, cells understand and use the intrinsic physico chemical aspects of actin self-assembly.

The rate of elongation in propulsive processes displays a bell-shaped dependence on CP concentration [35]. As discussed above, an increase in CP increases the rate of growth of uncapped ends. However, excess of CP blocks growth at all barbed ends, slowing down actin-based motile processes, eventually abolishing treadmilling.

Direct competition for barbed ends takes place between soluble CP and other barbed-end binding proteins like membrane-activated formins. This competition, which develops through so far unexplored mechanisms, is important in regulating the number of formin-initiated filaments in filopodial bundles, and the length and morphology of these bundles. The presence of a punctate pattern of CP along the filopodia and the rod-like appearance of filopodia in CP-depleted cells, contrasting with the tapered shape of filopodia in control cells [32], altogether testify that CP regulates not only the rate of extension of filopodia, but also the number of formin-attached filament barbed ends at the tip of filopodia.

The interplay between cappers and positive regulators of barbed end growth has drastic effects when the extent of barbed end capping varies within the narrow range of 95–100 %, in which a large change develops in the steady-state concentration of both polymerizable monomeric actin and sequestered (unpolymerizable) actin [7, 33, 36–38]. In other words, uncapping of only a few percent of filaments elicits massive effects on motility based on actin assembly. The predominant mass of soluble CP blocking barbed ends in the bulk cytoplasm ensures the establishment of a high monomer concentration required for local efficient barbed end growth of the transiently non-capped barbed ends, in lamellipodia or filopodia. Noteworthily, proteins harboring uncapping CPI motifs [27] like CARMIL, CIN85/CD2AP, FAM21, CAP-ZIP are all conspicuously associated with WASP protein-Arp2/3 mediated branched filament arrays. These regulators of CP dynamics at filament barbed ends localize at specific membrane-bound sites, ensuring localized effects on the dynamics and morphology of dendritic arrays of actin filaments.

As discussed earlier [39], de novo assembly of filaments from the pool of sequestered actin takes place as a relaxation process from one steady-state level to another one, upon a stimulus-induced change in reactivity of the barbed ends. The time dependent lowering of the steady-state free G-actin concentration is simultaneously amplified in mass by sequesterers, causing an increase in mass of F-actin equal to the decrease in mass of sequestered actin.

In conclusion, the regulation of barbed end capping, both in the bulk cytoplasm and at specific loci, is of crucial importance in motility.

## Interplay at barbed ends: mutually exclusive or non-competitive binding and “uncapping”

As briefly outlined in the introduction, if two regulators share only partial binding subsites on terminal subunits, they may bind the barbed end simultaneously, thus increasing the structural and functional complexity. Transient binding of the two proteins together may also facilitate and lead to the dissociation of one of them by the other. Typical physiologically relevant cases concern the regulation of the dwell time at barbed ends of proteins that by themselves dissociate very slowly, like CP and formins.

### Mechanisms of uncapping of CP

CP binds barbed ends with high affinity (Kd = 0.1 nM) and by itself dissociates from barbed ends with a half-time of about 30 min [40]. Both the abundance of CP and its extremely slow dissociation call for a regulation of its activity. This regulation is implemented either by controlling the availability of free CP or by enhancing its dissociation. Myotrophin V1 and CARMIL are two known regulators of CP that employ these mechanisms. Myotrophin simply sequesters free CP while CARMIL and proteins of the CapZIP family, which harbor a consensus CPI (capping protein interaction) motif, actually “uncap” CP from barbed ends via formation of a transient barbed end-bound CP–CARMIL complex ([24, 27, 41] for review).

Uncapping of CP was also shown to be mediated by dimerized WH2 domains, present in VopF/VopL or in Ena/VASP proteins. The *Vibrio cholerae*/*Vibrio parahaemolyticus* outer proteins VopF/VopL harbor a unique organization in dimers of three WH2 repeats. This feature is responsible for their ability to uncap actin filaments from barbed end-bound capping protein [21]. The barbed face of the terminal subunit of CP-capped barbed ends is occupied by the β-tentacle of CP [42, 43] (Fig. 3a, c) leaving the WH2-binding site largely available on the penultimate subunit. Therefore, VopF (V) and CP (C) can bind together to filament barbed ends (B), causing reduced affinity of both ligands, in a ternary complex BVC. Stabilization of VopF at barbed ends is provided by weak interactions of the C-terminal dimerization domain with the filament side. Rapid dissociation of CP from the transient BVC complex allows VopF to potentially track filament barbed ends in the BV state, possibly via alternate interactions of WH2 domains from opposite protomers with barbed end terminal subunits. Importantly, only 10 % uncapping has massive effects on barbed end reactivity [38]. VopF was proposed as a model for proteins of the Ena/VASP family, which consist of tetramerized WH2 domains adjacent to an F-actin-binding domain, and may track barbed ends using the same mechanism [44].

### Interplay of spire and formin 2 at barbed ends

Two other barbed end binding proteins, formin 2 and the four-WH2 repeat containing protein Spire, together regulate barbed ends in cytoplasmic actin assembly in asymmetric meiotic cell division. As compared to other formin FH1–FH2 domains, formin 2 by itself nucleates poorly in the presence of profilin–actin and binds extremely slowly to barbed ends. Spire, on its own,  caps barbed ends via its WH2 domains. Via association of its KIND domain with the C-terminal region (tail) of formin 2, Spire rapidly recruits formin 2 to the barbed end. This local interaction leads to rapid displacement of Spire by formin 2 at the barbed ends and onset of fast processive assembly [20] by formin 2. In other words, Spire behaves as an activator of the Rho-independent formin 2. This “kick-off” mechanism implies that binding of formin 2 to some of its available binding subsites at barbed ends weakens the binding of the WH2 domains of Spire, facilitating their competitive displacement by the homolog binding elements of formin 2. This mechanism is at the origin of the synergy between formin 2 (Cappuccino in *Drosophila*) and spire in mediating the massive assembly of cytoplasmic actin networks, which is required for spindle translocation to the cortex in mammalian asymmetric meiotic division, and for axis patterning at mid-oogenesis of *Drosophila* [45–47].

### Interplay of CP and formins at barbed ends

Formins and CP are considered as strict competitors for barbed end binding with formins “protecting” the barbed ends from CP ([27, 48] for review). However, CP caps formin-bound barbed ends, pending a greatly reduced affinity of CP [13], in the process causing rapid arrest of formin-mediated motile processes. Gelsolin also arrests the movement of formin coated beads and poisons them, consistent with formation of a stable complex at the bead surface between formin, actin and gelsolin [13]. The effects on barbed end dynamics which result from the transient association of both CP and formin at the same barbed end have bearings in the regulation of formin-mediated fast processive growth of filaments in vivo, and are being explored in full detail (Shekhar et al., submitted).

## Establishment and maintenance of polarity in actin assembly

Establishment and maintenance of polarized growth of filaments relies on transient or permanent links between the barbed ends of growing filaments and the membrane. As illustrated in Fig. 4, filament barbed ends appear to terminate at a membrane in lamellipodia protrusion, filopodia extension, focal adhesions and podosomes, endosomal fission and in pathogen propulsion. A large variety of “nucleators” of polarized actin assembly are at work in each of these processes. The links between the actin filament barbed ends and the membrane are thus structurally and temporally different in each of these individual cases that generally fall in the class of “site-directed barbed end assembly”. Using various molecular mechanisms, the interaction of the immobilized assembling machinery with the filament barbed ends allows filament growth from G-actin or profilin–actin, as well as the binding of competing soluble regulators like capping proteins. Site-directed barbed end assembly is thus controlled in a signal-responsive fashion at the membrane. Recent data indicate that the curvature of the membrane, linked to its lipid composition and association with BAR domain containing proteins, adds further complexity to the structure–function relationship at work in various shape changes [49–51].

### Formins as catalysts of insertional actin assembly

Formins are dimeric proteins that both nucleate actin assembly and catalyze rapid processive assembly of actin filaments. Formin-bound barbed ends elongate between 3- and 10-fold faster than unliganded barbed ends, and the formin often remains bound to the elongating barbed end for several minutes [12, 13]. Although the essential actin-binding moiety is the Formin Homology 2 (FH2) head-to-tail ring shaped dimer, the Formin Homology 1 (FH1) proline rich stretch binds profilin, which is required for the biological function of most formins. In vitro, profilin is required for fast processive barbed end assembly of filaments by formins. Hence, profilin–actin is the actual substrate of formin. The isolated FH2 ring structure in itself encircles barbed end terminal subunits in a conformation called the “closed” state, that either slows down or blocks barbed end growth (Fig. 3b, c) ([52–54], for review). Nucleation is facilitated energetically by stabilization of prenuclei actin dimers by the FH2 domain [55]. The additional association of the FH1 domain with profilin allows FH1–FH2 to bypass inhibition of nucleation by profilin and catalyze rapid processive assembly from profilin–actin [12, 13] (Fig. 4). The detailed structural changes at the formin-F-actin interface and related changes in binding strength of formin accompanying their tracking of growing barbed ends are not known ([52] for review). Actual rotation of formin around the elongating filament has been reported [56]. Formins indirectly cooperate with ADF/cofilin and cappers, which together increase the stationary pool of profilin–actin, used by formin in catalytic processive assembly [13].

Formins generally are activated by signaling molecules at membranes to promote insertional processive assembly. The available space between barbed ends and the membrane is potentially regulated chemically and mechanically, as described below.

### Formins are mechanosensitive machines

Due to their attachment to membranes, formins are able to sense and to react to forces coming from membrane tension, and they can apply a pulling force on elongating actin filaments. Quantitative measurements indicate that piconewton pulling forces applied by a microflow to a formin-bound filament accelerate the rate of processive assembly, and slow down processive disassembly [57]. These results have demonstrated that formins are mechanosensitive, and traction forces affect the biochemical cycle of formin tracking barbed ends. During the cycle of processive assembly, the FH2 domains of each protomer of the formin dimer are thought to alternate between a “closed”, strongly barbed end-bound state and an “open”, less strongly bound state [52, 54]. Consistently, pulling on barbed end-bound formins favors the “open” state of the FH2 domain in which weakened binding allows association of G-actin to the barbed end. In turn, slower disassembly under force reflects the lower percent of time spent by formin in the closed state in which disassembly is favored. Formins work under tension in many motile processes in vivo. For instance, in cytokinesis, formins initiate assembly of actin bundles at nodes, and have to work under traction forces exerted by myosin during closure of the cytokinetic ring. Formins may also work against compressive forces coming from membrane tension in protrusive or adhesive structures.

### Regulation of the length of formin-assembled filaments

The slow dissociation of formins from the barbed end during processive assembly can lead, even in the presence of a low concentration of profilin–actin (0.1 µM), to filaments of up to 20 µm in length before filament detachment from formin occurs. In bundles of filaments initiated by clustered formins, a fraction of formins remains bound to the bundle at any given time, increasing the period of time spent by the bundle in the attached state and the resulting bundle length. In vivo, however, the length of filopodia or of the actin filaments that compose the cytokinetic ring appears to reach only a limited length, suggesting that some down-regulation of processive assembly occurs. Bud14 has been identified as such a displacement factor that kicks off yeast formin Bnr1 from the barbed end [58]. Further association of Bud14 with Kelch proteins, homologs of fission yeast proteins tea1p and tea3p, regulates the length of formin-induced actin bundles in a large number of processes [59].

Similarly, capping proteins [13] and Cytochalasin D [60] rapidly displace formins from the barbed ends, causing detachment of actin bundles from formin-beads or aggregates. This result contrasts with the conventional view according to which formins “protect” barbed ends from their blockage by cappers. The structural details of the catalytic step at which displacement of formin by capping protein may be facilitated and the possible role of ATP hydrolysis are not yet known.

### WASP family proteins as membrane-bound catalysts of filament branching

Among the site-directed nucleators of filaments, the WASP family proteins (Table 2) are particularly interesting. These proteins are widespread and all catalyze the same reaction i.e., filament branching with Arp2/3 complex, in various cellular processes.

**Table 2:** WASP family proteins and related regulators

Proteins	In vivo functions	Cellular localization	Regulators	References
N-WASP	Formation of invadopodia and podosomes, internalization of endocytic vesicle	Filopodia	Cdc42, PIP2	[64–66]
WASH	Scission of tubular membranes in vesicular trafficking	Endosome	PIP3	[73, 74]
WHAMM/JMY	Drive Golgi reorganization	Golgi membrane, perinuclear region Brain tissue, neuronal cells	–	[75, 76]
SCAR/WAVE	Extension of lamellipodia	Lamellipodia	PIP3, IRSp53	[1]
WAFL	Endocytosis, formation of filopodia	Early Endosome	–	[119, 121]
DIP/WISH/SPIN90	Endocytosis, formation of lamellipodia	Lamellipodia	Cdc42, RhoA, Rac1	[77–79]
RickA (*Rickettsia*)	Infection of cell, use the actin-based motility system	Cytoplasm	–	[118, 120]
ActA (*Listeria*)	Infection of cell, use the actin-based motility system	Cytoplasm	–	[84, 85]

SCAR/WAVE proteins generate branched filament arrays to promote extension of lamellipodia [1] as well as cortical actin re-assembly in blebs [61] and many other developmental processes and synaptogenesis [62, 63]; N-WASP based branching is involved in formation of invadopodia and podosomes, internalization of endocytic vesicle [64–66], formation of dendritic spines [67, 68] and formation of cell–cell junctions mediated by cadherin [69–72]; WASH-induced filament branched arrays promote scission of tubular membranes in vesicular trafficking [73, 74]; WHAMM/JMY drives Golgi reorganization [75, 76] and DIP/WISH/SPIN90 proteins act in endocytosis and lamellipodia [77–79]. In these functions, WASP proteins all localize at membranes where they are activated and regulated by different mechanisms ([80–83], for reviews).

Central to the mechanism of force production is the catalysis of filament branching. Activation of WASP proteins always results in exposure of the C-terminal catalytic domain, called VCA or WCA. This domain consists of a WH2 motif (in one or two repeats), C (connector) and A (acidic) regions stand adjacent to each other in the sequence of most WASP proteins except WASH in which 100 residues separate C from A [83]. Incidentally, the *Listeria* ActA protein, which catalyzes filament branching responsible for *Listeria* propulsion in the host cytoplasm, harbors motifs structurally and functionally analogous to the WH2, C and A motifs of WASP proteins, but in different order in the sequence [84, 85].

At each catalytic cycle of filament branching, one molecule of Arp2/3 is incorporated at a branch junction from which a “daughter” filament is initiated (Fig. 4). Fluorescence imaging of the branched filament array reveals that filaments are created by branching at the very tip of the lamellipodium, and Arp2/3 and actin treadmill at identical rates through the meshwork [3], while WASP undergoes slow turnover at the membrane [86]. Reconstituted motility assays similarly show that Arp2/3 and actin are incorporated at the same rate into the dendritic meshwork growing from the bead at which N-WASP is immobilized [35]. When vesicles are functionalized with N-WASP, co-segregation of actin and N-WASP takes place during propulsion [87]. In conclusion, filament growth and site-directed branching are kinetically coupled reactions. Branching creates a transient link between individual actin filaments and the membrane, following which the filament barbed ends grow transiently, developing a pushing force while keeping their barbed ends oriented toward the membrane (Fig. 4). At the macroscopic level of the dendritic meshwork, this mechanism is equivalent to processive assembly, since branching maintains a fraction of the newly formed filaments attached during assembly of the network, as described in the tethered ratchet model [88]. Delayed barbed end capping, debranching and pointed end disassembly, together maintain the stationary morphology of the array.

The general topology, high resolution structure and dynamics of the large macromolecular complex formed at the membrane between a filament, WASP and Arp2/3 in the catalysis of branching represent a major challenge for future research.

### Kinetic analysis of assembly of branched filaments in solution

The isolated VCA has been extensively used to analyze filament branching both in bulk solution and in single filament TIRF microscopy assays. Polymerization of actin in the presence of VCA and Arp2/3 complex shows constant acceleration due to autocatalytic multiplication of growing filaments by branching. Addition of pre-assembled filaments at time zero shortens the initial acceleration period, in a manner dependent on the number of barbed ends rather than on the mass of added F-actin. This feature is suggestive of barbed end branching by Arp2/3. It is further consistent with the measured length correlation of mother and daughter filaments [7]; Contradictory data, obtained using capped filaments to stimulate branching, leave open the issue of filament side versus barbed end branching [84, 89].

TIRF microscopy was used to visualize live assembly and branching of single filaments anchored by myosin or maintained in close proximity of the glass surface [90]. In this 2D geometry, branches emerge from the sides of filaments, and events compatible with side and end branching are seen as well [91, 92]. Side-branching appeared favored on the convex face of curved filaments [93]. High resolution single molecule imaging and quantitative analysis of branching by VCA and Arp2/3 complex show that binding of Arp2/3 to filament sides is slow, association is two- to fourfold faster with VCA, although quite slow (k+ = 0.025 µM^−1^ s^−1^ per F-actin subunit); growth of a daughter branch occurs extremely infrequently, from only 1 % of the filament-bound Arp2/3 [94], and is kinetically limited by dissociation of VCA [95]. The remarkably low efficiency of side-branching in 2D microscopy assays contrasts with the densely branched actin meshwork observed in lamellipodia, in actin tails of pathogens and in 3D bulk solution polymerization assays.

### Biochemical and structural analysis of complexes of Arp2/3 with VCA, monomeric actin and actin filaments

Biochemical and structural analysis of the interactions between VCA, actin and Arp2/3 complex is at the heart of the possible mechanisms of filament branching. Binding of Arp2/3 to VCA induces a structural change in Arp2/3 complex that strengthens binding of ATP to Arp2 [96]. The Arp2 subunit is essential for filament branching by VCA-Arp2/3 [97, 98]. The WH2 domain (V) of VCA, even in absence of Arp2/3, binds G-actin in a complex that participates in filament barbed end assembly like profilin–actin [99, 100]. Via its WH2 domain VCA also captures filament barbed ends [101] and elicits rapid processive barbed end assembly [102]. The entire CA region interacts with Arp2/3 complex. Various structural studies of the branching complex and the filament branch junction so far fail to provide a comprehensive view of the interface between Arp2/3 and VCA, and the position of all subunits at the branch junction [103–106].

The VCA–actin–Arp2/3 complex, considered as the “branching complex” that interacts with the mother filament [107], displays a 1:1:1 stoichiometry in gel filtration [108]. A second VCA low affinity binding site (Kd = 1.6 µM) was detected on Arp2/3 complex [109, 110]. Its putative role in branching is hard to reconcile with the nanomolar range of efficiency of VCA and the fact that efficient propulsion of N-WASP functionalized particles is recorded when the N-WASP molecules are at a distance of 20 nm [35]. The limited information addressing how Arp2/3 complex branches filaments is an obstacle to understanding how regulators like CK666 [111] and glia maturation factor (GMF, a debranching factor of the ADF/cofilin family, [112]) act.

Technical difficulties that have slowed our progress include the limited available resolution in the structures of large macromolecular complexes, the limited available amounts of native and recombinant genetically modified Arp2/3 complex, and the 2D constraints imposed by TIRF microscopy in monitoring formation of the 3D structure of branched filaments. Due to these difficulties, the proposed models are dominated by assumptions made ab initio on structural changes in the organization of the Arp2/3 complex during branching.

### In vivo analysis of dendritic meshworks

The high density of the intricate array of branched filaments in the actin tail of propelling pathogens precludes the accurate tracking of individual filaments in electron micrographs [113, 114]. This limitation was overcome using the baculovirus, which propels by site-directed assembly of a small number of individually identified branched filaments [115]. The virus appears to move at a few µm/min using only a dozen of pushing filaments that are branched at the virus surface. The barbed ends of the individual newly assembled filaments are seen bound to the virus surface. Very similar EM observations have been made on 50 nm ActA-coated beads placed in cell extracts [6].

### Coordination between the turnover of WASP-Arp2/3 promoted branched filament arrays and formin-induced bundles that co-exist in cell processes

Assuming that a pool of polymerizable actin monomers is established and feeds site-directed barbed end elongation, an unsolved issue concerns the seemingly homogeneous rate of protrusion and filament turnover in the lamellipodia, where both dendritic filament arrays and formin-mediated filopodia and microspikes co-exist [116]. Formins are expected, under this condition, to mediate about five- to tenfold faster actin assembly than the free barbed ends. Yet the turnover of actin filaments appears maintained at the same value within all these structures, generating a smooth leading edge. A possible explanation is that the mechanical rigidity of the membrane imposes a load that regulates barbed end growth in lamellipodia. In contrast, dendritic filament arrays and filopodia segregate in dendritic spines and mediate different rates of protrusion [67], which may indicate that the tension of the membrane in these cellular extensions is lower, enabling more dynamic changes in shape.

## Conclusions and perspectives

The control of actin filament barbed end dynamics is mediated by a large number of soluble as well as membrane-bound effectors, which may act either directly by binding barbed ends, or indirectly by affecting the on flux of actin monomers at barbed ends.

Effectors that bind barbed ends may either exclude each other or bind in synergy together at the barbed ends, or may displace each other from the barbed end via formation of transient ternary complexes. The binding of regulatory proteins may further be affected by the state of the nucleotide bound to actin subunits at the barbed end. Many of these processes also include irreversible ATP hydrolysis. Elucidating such complex binding schemes, will require more extensive biochemical, kinetic, as well as structural studies.

High resolution electron microscopy of complexes formed at the barbed ends is anticipated to foster our progress. Rapid kinetics of the changes in reactivity of filament barbed ends using microfluidics-assisted TIRF microscopy of individual filaments, in assembly and disassembly regimes and presence of various regulatory ligands, is clearly an avenue for mechanistic studies.

Because most of the regulation of polarized filament assembly is mediated by regulators that are bound both to membranes and to filament barbed ends, the chirality of the growing helical filament plays an important role. A consequence of the chirality of the actin filament is revealed at high scale in the chiral organization of cytoskeletal patterns of radial and transverse fibers in fibroblasts constrained to a circular shape [117]. Future force-based approaches of the bearings of this intrinsic property of actin in morphogenetic processes include the application of controlled membrane tension and torque to the growing helical filament. New experiments will have to be designed to address these issues.
